# Early activation of nSMase2/ceramide pathway in astrocytes is involved in ischemia-associated neuronal damage via inflammation in rat hippocampi

**DOI:** 10.1186/1742-2094-10-109

**Published:** 2013-09-03

**Authors:** LiZe Gu, BaoSheng Huang, Wei Shen, Li Gao, ZhengZheng Ding, HuiWen Wu, Jun Guo

**Affiliations:** 1 Department of Biochemistry and Molecular Biology, Nanjing Medical University, Nanjing 210029, People’s Republic of China; 2 Department of Neurosurgery, BenQ Medical Center, Nanjing Medical University, Nanjing 210029, People’s Republic of China; 3 Department of Neurology, Brain Hospital Affiliated to Nanjing Medical University, Nanjing 210029, People’s Republic of China

**Keywords:** Astrocyte, Ceramide, Cytokine, Ischemia, nSMase2 protein, p38MAPK, Rat model

## Abstract

**Background:**

Ceramide accumulation is considered a contributing factor to neuronal dysfunction and damage. However, the underlying mechanisms that occur following ischemic insult are still unclear.

**Methods:**

In the present study, we established cerebral ischemia models using four-vessel occlusion and oxygen-glucose deprivation methods. The hippocampus neural cells were subjected to immunohistochemistry and immunofluorescence staining for ceramide and neutral sphingomyelinase 2 (nSMase2) levels; immunoprecipitation and immunoblot analysis for nSMase2, receptor for activated C kinase 1 (RACK1), embryonic ectoderm development (EED), p38 mitogen-activated protein kinase (p38MAPK) and phosphorylated p38MAPK expression; SMase assay for nSMase and acid sphingomyelinase (aSMase) activity; real-time reverse transcription polymerase chain reaction for cytokine expression; and Nissl, microtubule-associated protein 2 and terminal deoxynucleotidyl transferase–mediated deoxyuridine triphosphate nick-end labeling staining.

**Results:**

We found considerable production of ceramide in astrocytes, but not in neurons, during early cerebral ischemia. This was accompanied by the induction of nSMase (but not aSMase) activity in the rat hippocampi. The inhibition of nSMase2 activity effectively reduced ceramide accumulation in astrocytes and alleviated neuronal damage to some extent. Meanwhile, the expression levels of proinflammatory cytokines, including tumor necrosis factor α (TNF-α), interleukin 1β (IL-1β) and IL-6, were found to be upregulated, which may have played an import role in neuronal damage mediated by the nSMase2/ceramide pathway. Although enhanced binding of nSMase2 with RACK1 and EED were also observed after cerebral ischemia, nSMase2 activity was not blocked by the TNF-α receptor inhibitor through RACK1/EED signaling. p38MAPK, but not protein kinase Cζ or protein phosphatase 2B, was able to induce nSMase2 activation after ischemia. p38MAPK can be induced by A2B adenosine receptor (A_2B_AR) activity.

**Conclusions:**

These results indicate that the inhibition of ceramide production in astrocytes by targeting A_2B_AR/p38MAPK/nSMase2 signaling may represent a viable approach for attenuating inflammatory responses and neuronal damage after cerebral ischemia.

## Background

Ischemic stroke, usually induced by a temporary or permanent reduction of local cerebral blood flow, is the leading cause of death and disability in humans [1-5]. Among the several molecular mechanisms that contribute to ischemia-induced brain damage, inflammation plays a crucial role. It not only is involved in the pathophysiology of cerebral ischemia but also is recognized as being a risk factor for ischemic insult. Recent studies suggest that astrocytes are involve vitally in neuroinflammation through the release of cytotoxic agents in response to ischemia stimuli, which can contribute to blood–brain barrier disruption and delayed neuronal damage [6-8]. However, the mechanisms underlying inflammation in astrocytes after ischemia remain unclear.

Ceramide, a liposoluble messenger, has been identified as an important signaling molecule in cell growth arrest, differentiation, senescence, apoptosis and inflammation [9-13]. After ceramide generation, a number of signaling molecules, including c-Jun N-terminal kinase (JNK), protein kinase Cζ (PKCζ), stress-activated protein kinase, kinase suppressor of Ras, protein phosphatase 1 (PP1) and PP2A, can be activated to regulate the intracellular signaling pathway and induce a stress reaction in the target cells [14-17]. Our studies found that in the early stage of acute brain ischemic injury, specific accumulation of ceramide was notable in hippocampus astrocytes but not in neurons, which might be associated with neuroinflammation in the postischemic brain tissue [18,19].

In many cases, sphingomyelin (SM) hydrolysis by sphingomyelinase (SMase) appears to be a major, rapid source of ceramide production in response to various conditions, such as hypoxia, oxidative stress and ultraviolet irradiation [20-22]. SMase can be divided into several subtypes according to different pH optima and subcellular localizations, such as neutral sphingomyelinase (nSMase) localized in the plasma membrane and acid sphingomyelinase (aSMase) localized in endosomal-lysosomal compartments [23,24]. Among the subtypes of nSMase, nSMase2 is considered to be an important candidate for ceramide production in the neural membrane [25,26].

nSMase2 activation is closely associated with its WD repeat domain and the phosphorylation of special sites [25]. For example, the activity of nSMase2 can be stimulated by tumor necrosis factor α (TNF-α) through the formation of a WD-binding complex (TNF-R1-FAN-RACK1-nSMase2) [27,28]. In addition, it has recently been reported that embryonic ectoderm development (EED) may be the last missing link between receptor for activated C kinase 1 (RACK1) and nSMase2 [29]. Moreover, nSMase2 is also recognized as a phosphoprotein with five highly conserved serine residues (S173, S208, S289, S292 and S299), and its activity can be regulated by kinases and phosphatases in response to certain stresses [30,31]. p38, PKCδ and PP2B have been recognized as being upstream of nSMase2, which can regulate its activity through serine phosphorylation and dephosphorylation [32-34]. Moreover, p38 pathways have been found to be involved in the A2B adenosine receptor (A_2B_AR)-mediated inflammatory response [35,36].

The four-vessel occlusion (4-VO) procedure is widely used to induce forebrain ischemia and cause delayed neuronal death in the rat hippocampus, especially in its CA1 region, similarly to the clinical rationale in ischemic stroke. During cerebral ischemia, ceramide production is thought to be closely associated with neuron damage in the hippocampal region [8,9]. Astrocytes are now recognized as innate immunocytes which possess the potential to release various kinds of inflammatory mediators [37,38]. Therefore, we propose that cerebral ischemia can stimulate nSMase2-induced SM hydrolysis and ceramide production in astrocytes, which is followed by the production and release of inflammatory mediators from activated astrocytes. These inflammatory mediators in turn act on neurons and aggravate secondary damage of neurons in the central nervous system.

## Methods

### Animal model of ischemia

All animal experiments were performed in accordance with the Guide for the Care and Use of Laboratory Animals of the National Institutes of Health and approved by the Institutional Animal Care and Use Committee of Nanjing Medical University, China. Adult male Sprague–Dawley rats (purchased from the Experimental Animal Center of Nanjing Medical University) weighing 220 to 250 g were used in the study.

The method of inducing transient global ischemia was performed as described previously [39,40]. All animals (except the sham controls) underwent 4-VO. Briefly, the animals were anesthetized with 10% chloral hydrate (350 mg/kg intraperitoneal injection), then the vertebral arteries were occluded by electrocautery. On the following day, 4-VO ischemia was induced for 10 min by occluding the bilateral common carotid arteries with aneurysm clips. Animals that lost their righting reflex within 30 s and whose pupils were dilated and unresponsive to light were used for the subsequent experiments. After 10 min of ischemia, the clips were removed for reperfusion. The animals in the sham group underwent the same surgical procedure; however, the carotid arteries were only exposed and not occluded. During the experiment, the rats’ body temperature was maintained at around 36.5°C.

### Infusion and administration of drugs or small interfering RNA

The drugs or their vehicles were injected into the lateral ventricles (0.8 mm posterior and 1.5 mm lateral to the bregma, 3.5 mm deep) using a microinjector 30 min before the induction of ischemia, as described in previous reports [39,40]. The compounds used are listed in Table 1.

**Table 1:** **Signal transduction inhibitors or agonists and their corresponding targets**^**a**^

**Agent**	**Abbreviation**	**Target**	**Company**
GW4869		nSMase2 inhibitor	Santa Cruz Biotechnology
Daunorubicin	DNR	nSMase2 agonist	
R-7050		TNF-α receptor antagonist	
SB-203580		p38MAPK inhibitor	
PP2B inhibitor dithiocarbamate		PP2B inhibitor	
Imipramine	Lim	aSMase inhibitor	Sigma-Aldrich
MRS-1754		Selective antagonist for A2B adenosine receptors	
Rottlerin		PKCδ inhibitor	Enzo Life Sciences

^a^*aSMase* acid sphingomyelinase, *nSMase2* neutral sphingomyelinase 2, *p38MAPK* p38 mitogen-activated kinase, *PKCδ* protein kinase Cδ, *PP2B* protein phosphatase 2B, *TNF-α* tumor necrosis factor α.

For the administration of small interfering RNA (siRNA), 5 μl of control siRNA (a scrambled siRNA) or nSMase2 siRNA (20 μM; RiboBio Co, Guangzhou, China) were diluted with the same volume of transfection reagent (Santa Cruz Biotechnology, Santa Cruz, CA, USA). The injection was repeated four times, every 12 h, starting 2 days before ischemia induction, as described previously [40]. After injection, the needle was kept in place for 5 min.

### Isolation of primary rat neurons and astrocytes

Under sterile conditions, the hippocampi were dissected from neonatal rats on postnatal day 1 and then dissociated by trituration and trypsinization (0.25% trypsin in Dulbecco’s modified Eagle’s medium (DMEM) at 37°C for 15 min). Digestion was terminated with 10% fetal bovine serum (FBS) (Gibco/Life Technologies, Grand Island, NY, USA), then the tissues were filtered through 200-μm mesh. The samples were centrifuged at 5,000 *g* for 5 min. Primary rat neurons were cultured in neurobasal medium with 2% B27 supplement and 1% antibiotic-antimycotic mixture (Gibco/Life Technologies) at 37°C in a 5% CO_2_ atmosphere. At the same time, the primary rat astrocytes were cultured in DMEM with 10% FBS at 37°C in a 5% CO_2_ atmosphere.

### Oxygen-glucose deprivation model

Before exposure to oxygen-glucose deprivation (OGD) conditions, the culture medium was changed to glucose-free DMEM without serum as described in previous reports [41,42]. The astrocytes were exposed to 0.1% O_2_, 5% CO_2_ and 94.4% nitrogen for 3 h or 6 h at 37°C, then they were returned to the culture medium with glucose and serum supplement for 30 min at 37°C in a 5% CO_2_ atmosphere.

### Immunohistochemistry and immunofluorescence

Rats were perfused with 0.9% saline and 4% paraformaldehyde. The brains were frozen, sectioned (20 μm) and blocked with 3% bovine serum albumin (BSA) (0.1% Tween 20) for 30 min at 4°C. The immunohistochemistry samples were incubated for 10 min with 1% H_2_O_2_ and then blocked. The sections were incubated with primary antibodies, including nSMase2 (1:200; Santa Cruz Biotechnology), ceramide (1:200; Sigma-Aldrich, St Louis, MO, USA), glial fibrillary acidic protein (1:500; EMD Millipore, Billerica, MA, USA) and NeuN (1:500; Millipore), for 24 h at 4°C. The slides were further examined using secondary antibodies labeled with tetramethylrhodamine isothiocyanate, fluorescein/rhodamine isothiocyanate or horseradish peroxidase (HRP). Finally, the immunohistochemistry stained sections were incubated with 3,3′-diaminobenzidine (DAB) reagent. Images were captured using a fluorescence microscope and analyzed using ImageJ software (National Institutes of Health, Bethesda, MD, USA).

### Nissl staining

Sections mounted on poly-L-lysine-coated slides were dehydrated with ethanol and then treated with xylene for 5 min. After being washed with double-distilled water, the sections were incubated with 1% cresyl violet (Sigma-Aldrich) solution for 5 min at 50°C and then dehydrated with ethanol. Images were captured using a visible microscope objective.

### Coimmunoprecipitation and immunoblotting

The hippocampi were dissected and harvested in lysis buffer containing a protease inhibitor cocktail (Sigma-Aldrich), 50 mM Tris•HCl, 150 mM NaCl, 1% Triton X-100, 1 mM ethylenediaminetetraacetic acid, 1 mM phenylmethylsulfonyl fluoride, 1 mM NaF and 1 mM NaVO_4_ (pH 7.4). The same amounts of the lysates were incubated with 40 μg of nSMase2 antibody (Santa Cruz Biotechnology) overnight at 4°C. The protein A agarose sphere (Santa Cruz Biotechnology) was added to the samples and stored at 4°C. After 2 h, the samples were washed three times with lysis buffer, and the immune complexes were collected. Part of the immunoprecipitation (IP)-purified nSMase2 was prepared for activity analysis, and another part was eluted using Laemmli buffer with 5% mercaptoethanol, before being boiled for 10 min. Anti-RACK1 (1:500; Cell Signaling Technology, Danvers, MA, USA) and anti-EED (1:500; Santa Cruz Biotechnology) antibodies were used for immunoblotting.

Denatured samples were separated by 10% SDS-PAGE and then electrotransferred onto a nitrocellulose membrane. After being blocked for 3 h, membranes were incubated with primary antibodies, including nSMase2 (1:500; Santa Cruz Biotechnology), RACK1 (1:500; Cell Signaling Technology), EED (1:500; Santa Cruz Biotechnology), p38MAPK (1:500; Santa Cruz Biotechnology), phosphorylated p38MAPK (1:1,000; Cell Signaling Technology) and β-actin (1:1,000; Santa Cruz Biotechnology) overnight at 4°C. The immunocomplex was also left to react with HRP-conjugated secondary antibodies. Finally, the signals on membranes were analyzed using the Jieda Image Analysis System (Jiangsu Jeda Science*-*Technology Co Ltd, Nanjing*,* China).

### Acid and neutral sphingomyelinase enzyme activities

SMase activity was analyzed using the Amplex Red Sphingomyelinase Assay Kit (Invitrogen/Life Technologies, Carlsbad, CA, USA). Briefly, the total protein (50 μg) was mixed with enzyme assay buffer (100 μl; pH 7.4) and added to a 96-well microtiter plate. The working solution, which contained choline oxidase (0.2 U/ml), alkaline phosphatase (8 U/ml), HRP (2 U/ml), Amplex Red reagent (0.1 mM) and SM (0.5 mM), was mixed in each well. The 96-well plate was incubated for 1 h at 37°C. Exposure to light was avoided. The Amplex Red reagent reacts to generate the specific fluorescent product, which was measured using the fluorescence plate reader at 571-nm excitation and 585-nm emission. The assay mixture for aSMase contained 0.1 mM acetate buffer (pH 5.8). The activity of nSMase2 was assessed using the Amplex Red Sphingomyelinase Assay Kit as described in previous reports [43]; however, the sample was the IP-purified enzyme (about 5 μg in 100 μl of elution buffer), not the total protein.

### RNA extraction and quantitative real-time polymerase chain reaction

Total RNA was isolated from hippocampal tissue using TRIzol reagent (Invitrogen, Carlsbad, CA, USA) according to the manufacturer’s instructions. Reverse transcription was performed using the PrimeScript RT Reagent Kit (TaKaRa Biotechnology, Dalian, China) according to the manufacturer’s protocol. The expression levels of the mRNA were analyzed using the SYBR *Premix Ex Taq* real-time quantitative PCR kit (TaKaRa Biotechnology) according to the manufacturer’s instructions. Real-time PCR was performed using the Eppendorf MasterCycler RealPlex Sequence Detection System (Life Technologies, Grand Island, NY, USA). Data analysis was performed using the 2^-ΔΔCT^ method.

### Astrocyte/neuron Transwell study

Primary rat astrocytes were cultured on permeable membranes using Millicell cell culture inserts (0.4-mm pore size; EMD Millipore) in six-well plates for 2 days at 37°C in a 5% CO_2_ Atmosphere. After 24 h of stimulation with the nSMase2 agonist daunorubicin (DNR; 0.5 μM), the inserts were placed onto the wells containing primary rat neurons. In this Transwell (Corning, Tewksbury, MA, USA) model, neurons were in the lower chambers facing each other, and astrocytes were kept independent in the upper chambers. Following the independent analysis of neuronal and glial groups, the soluble factors released from activated astrocytes could act upon the primary rat neurons in the lower chambers.

### Microtubule-associated protein 2 staining

Primary rat neurons in coverslips were fixed for 10 min at room temperature in 4% paraformaldehyde. After fixation, neurons were washed three times, treated with phosphate-buffered saline (PBS) plus 1% Tween 20 for 10 min at room temperature and blocked using 4% BSA. Staining for microtubule-associated protein 2 (MAP2) was performed using a rabbit anti-MAP2 antibody (1:200; Bioss, Woburn, MA, USA) for immunofluorescence as described above, then treated with 4′,6-diamidino-2-phenylindole stain (Invitrogen/Life Technologies).

### TUNEL assay

The terminal deoxynucleotidyl transferase–mediated deoxyuridine triphosphate nick-end labeling (TUNEL) assay was performed using the In Situ Cell Death Detection Kit (KGA702; KeyGEN Biotech, Nanjing, China) according to the manufacturer’s instructions. Briefly, after being permeabilized with 0.1% PBS-Triton X-100 for 5 min and blocked with 3% H_2_O_2_ for 10 min, the slides were incubated with TUNEL reaction mixture, including equilibration buffer, biotin-labeled deoxyuridine triphosphate (Biotin-11-dUTP) and terminal deoxynucleotidyl transferase enzyme, for 1 h at 37°C. The neurons were treated with streptavidin-HRP for 30 min at room temperature and incubated with DAB reagent.

### Data analysis

All data are expressed as the mean ± SD values from at least four animals. Statistical analysis was conducted using one-way analysis of variance followed by the Newman–Keuls test. Comparisons between the two groups were performed using Student’s *t*-test. *P* values <0.05 were considered significant.

## Results

### Ceramide induced by cerebral ischemia accumulates in hippocampal astrocytes and is related to sphingomyelin hydrolysis

Studies have shown that some harmful factors in neurodegenerative diseases (such as Alzheimer disease) can stimulate nSMase to produce ceramide, inducing astrocyte activation, the release of neurotoxic molecules and neuronal damage [9,10,14,44]. To investigate whether the nSMase/ceramide pathway is involved in cerebral ischemia-reperfusion (I/R) regulation, we first established a forebrain ischemia rat model (4-VO). Immunohistochemistry and immunofluorescence double-staining were carried out to detect the morphological localization of ceramide in rat hippocampi.

**Figure 1:**
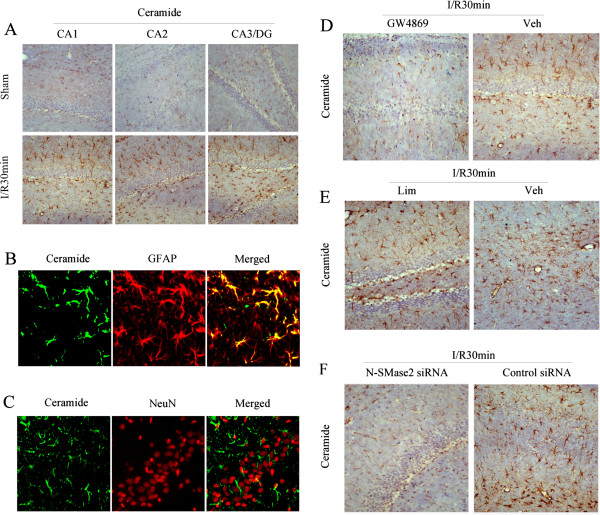
**Ceramide localization in rat hippocampus postischemia and effects of neutral sphingomyelinase 2 and acid sphingomyelinase inhibitors on ceramide. (A)** Ceramide levels in rat CA1, CA2 and CA3/dentate gyrus hippocampal areas were detected by immunohistochemistry in the ischemia-reperfusion (I/R) 30-min and sham control groups. **(B)** Double-staining of ceramide and glial fibrillary acidic protein in rat CA1 hippocampal area in the I/R 30-min group was detected using immunofluorescence. **(C)** Double-staining of ceramide and NeuN in rat CA1 hippocampal area in the I/R 30-min group was detected using immunofluorescence. Different drugs or small interfering RNA (siRNA) were injected into the lateral ventricle prior to the 10-min ischemia and 30-min reperfusion procedure. Drugs included GW4869 or its vehicle **(D)**, imipramine (Lim) or its vehicle **(E)** and neutral sphingomyelinase 2 siRNA or control siRNA **(F)**. Immunohistochemistry was subsequently carried out to detect ceramide expression levels (*n* = 4).

After 10 min of ischemia followed by 30 min of reperfusion, a considerable level of ceramide was found in CA1, CA2 and CA3/dentate gyrus (DG) hippocampal areas (Figure 1A), primarily in astrocytes but not in neurons (Figures 1B and C). As reported previously [23-26,44], SM hydrolysis can be an important means of rapidly generating ceramide. To further explore the molecular mechanism underlying ceramide accumulation induced by cerebral ischemia, inhibitor GW4869 and siRNA of nSMase2, and aSMase inhibitor imipramine (Lim; 10 μM), respectively, were injected into the cerebral ventricle prior to ischemia. The results indicated that ceramide levels in the hippocampus were decreased after treatment with GW4869 and nSMase2 siRNA (0.42-fold vs. vehicle or 0.47-fold vs. control siRNA; *P* < 0.05) (Figures 1D and F), but that there was no obvious change after Lim treatment (Figure 1E). Furthermore, the specificity of the staining was confirmed by replacement of the primary antibody with isotype-matched nonimmune immunoglobulin G or serum. Taken together, the results suggest that ischemia-induced ceramide accumulation was located specifically in rat hippocampal astrocytes. This might derive from SM hydrolysis by nSMase, especially nSMase2, but it has no connection with aSMase.

### Neutral sphingomyelinase 2 activity in astrocytes is quickly upregulated after cerebral ischemia

To confirm the speculation that nSMase (primarily nSMase2) might participate in the production of ceramide following I/R, a SM enzyme activity assay kit was used to examine the activities of nSMase, aSMase and nSMase2. In this study, the hippocampal tissues were extracted following different durations of cerebral I/R (0.5, 1, 6, 12 and 24 h).

**Figure 2:**
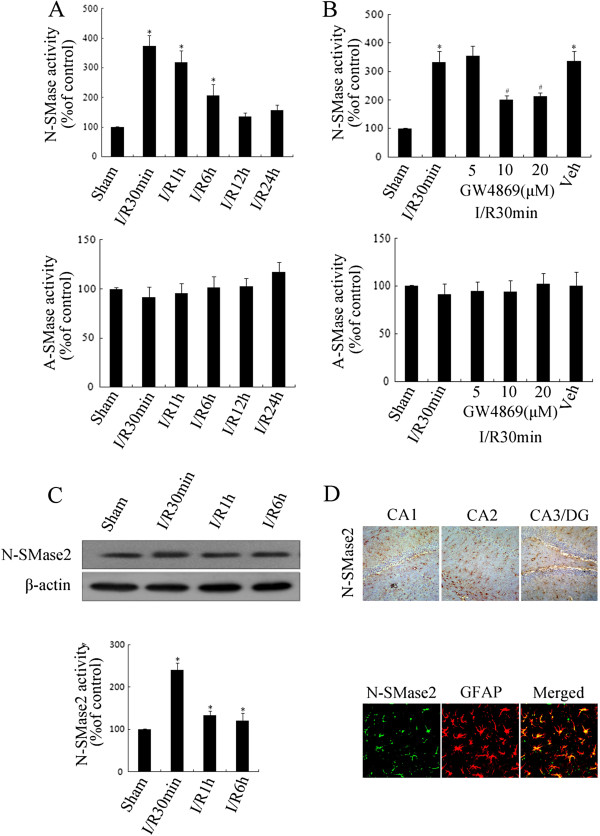
**Cerebral ischemia induced rapid activation of neutral sphingomyelinase and neutral sphingomyelinase 2 in rat hippocampal astrocytes. (A)** After postischemic reperfusion of different durations (30 min, 1 h, 6 h, 12 h and 24 h), neutral sphingomyelinase (nMase) or acid sphingomyelinase (aSMase) activity in the rat hippocampus was detected. **(B)** GW4869 and its solvent were separately injected into the lateral ventricle prior to the 30-min post-ischemia-reperfusion (post-I/R) procedure, and activity levels of nSMase and aSMase in the rat hippocampi were then measured. **(C)** After different durations of post-I/R (30 min, 1 h and 6 h), activity and protein expression levels of nSMase2 in the rat hippocampus were measured. **(D)** Examination of nSMase2 expression levels in rat CA1, CA2 and CA3/dentate gyrus hippocampal areas and double-staining of nSMase2 and glial fibrillary acidic protein in the CA1 area of normal groups were carried out by immunohistochemistry and immunofluorescence, respectively. Data are representative of the means ± SEM of four separate experiments (*n* = 4). *P < 0.05 vs. sham control. #*P* < 0.05 vs. ischemia solvent control.

As the time curve results show (Figure 2A), nSMase activity increased (compared with the control), and it reached 373.1% at I/R 30 min (*P* < 0.05), 317.6% at I/R 1 h (*P* < 0.05) and 246.2% at I/R 6 h (*P* < 0.05), but there were no differences for aSMase activity (*P* > 0.05). Moreover, GW4869 prompted considerable activity of nSMase (*P* < 0.05), but not of aSMase (Figure 2B), suggesting that nSMase2 might be the most important component of nSMase in the neural cells [25,26].

nSMase2 activity following cerebral ischemia was subsequently examined. nSMase2 protein was immunoprecipitated with a special antibody and analyzed using a SMase assay kit. nSMase2 activity at I/R 30 min, I/R 1 h and I/R 6 h all increased (*P* < 0.05), particularly at I/R 30 min. nSMase2 protein levels for each group were almost parallel, indicating that the difference was due to its own activity (Figure 2C). The protein was predominantly expressed in astrocytes in the CA1, CA2 and CA3/DG hippocampal areas (Figure 2D).

Taken together, the findings suggest that it is nSMase (mainly nSMase 2), not aSMase, that was initiated at the early stage of cerebral I/R and which played a significant role in the generation of ceramide in rat hippocampal astrocytes.

### Early initiation of neutral sphingomyelinase 2 induces neuronal damage associated with cerebral ischemia-reperfusion

**Figure 3:**
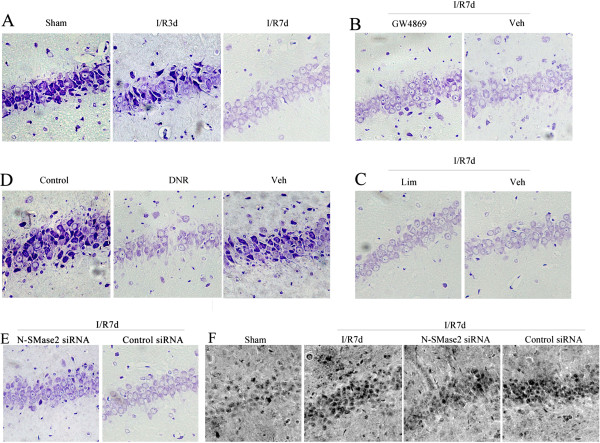
**Effect of neutral sphingomyelinase 2 on ischemic neuronal damage associated with cerebral ischemia.** Neuronal damage in the rat CA1 hippocampal area was detected in the different groups that received different treatments using Nissl staining. **(A)** Postischemic reperfusion of different durations (3 d and 7 d) and control groups. **(B)** and **(C)** Injection with GW4869, imipramine (Lim) and their solvent into the lateral ventricle, respectively, followed by 7-d postischemic reperfusion. **(D)** Normal group with daunorubicin (DNR) stimulus for 24 h. **(E)** Effect of neutral sphingomyelinase 2 (nSMase2) small interfering RNA (siRNA) and control siRNA on the rat CA1 hippocampal neurons postischemia. **(F)** Terminal deoxynucleotidyl transferase–mediated deoxyuridine triphosphate nick-end labeling assays showing apoptosis in the rat CA1 hippocampal neurons postischemia and effect of nSMase2 siRNA on them (*n* = 4).

Ceramide accumulation has been suggested to be an important factor in the development of ischemia lesions [9,10]. To determine the involvement of ceramide in neuronal injury associated with cerebral I/R, we utilized Nissl staining in the present study. Brain tissue was extracted at different times (3 d and 7 d) following cerebral I/R. Compared with the control group, the number of Nissl bodies in the neurons of rat hippocampi in the CA1 area was reduced at I/R 3 d. This effect was even greater at I/R 7 d (Figure 3A). However, neuronal damage was somewhat alleviated with GW4869 or nSMase2 siRNA treatment, but Lim had no effect (Figures 3B, C and E). This indicates the participation of nSMase2, but not aSMase, in neuronal damage induced by cerebral I/R. To explore this finding further, the nSMase2 agonist DNR (0.5 μM) was used [45], which revealed that the number of Nissl bodies was remarkably reduced by DNR (Figure 3D). In addition, the TUNEL assay results (similar to those revealed by Nissl staining) also suggested that nSMase2 had some involvement in the apoptosis of neurons post-ischemia (Figure 3F). Overall, it is suggested that the nSMase2/ceramide pathway in astrocytes was linked to the neuronal damage after cerebral ischemia in vivo.

### TNF-αR/RACK1/EED pathway partly mediates early initiation of neutral sphingomyelinase 2 during ischemia

**Figure 4:**
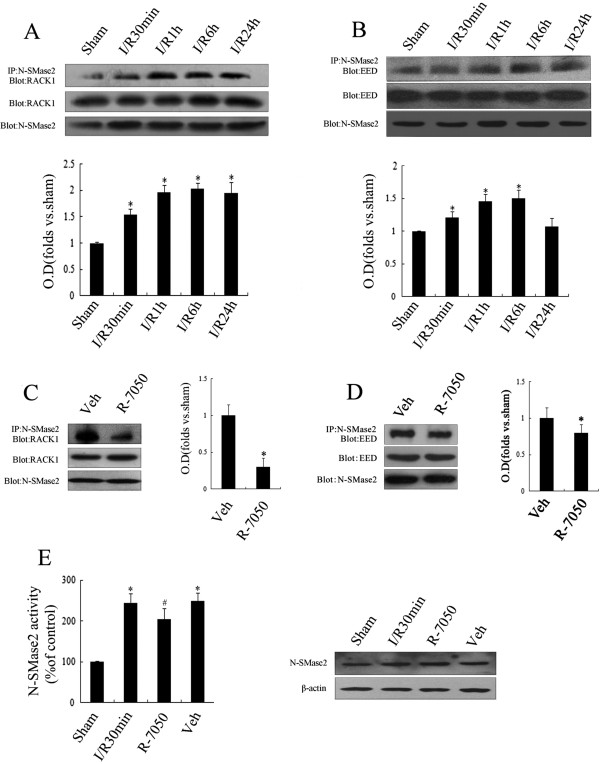
**Effect of the TNF-αR/RACK1 pathway on early activation of ischemia-induced neutral sphingomyelinase 2. (A)** and **(B)** The binding effects of neutral sphingomyelinase 2 (nSMase2) with receptor for activated C kinase 1 (RACK1) and embryonic ectoderm development (EED) were detected using coimmunoprecipitation after postischemic reperfusion of different durations (0.5 h, 1 h, 6 h and 24 h). **(C)** and **(D)** The binding effect of nSMase2 with RACK1 and EED was detected using coimmunoprecipitation after R-7050 or solvent was injected into the lateral ventricle with 30 min postischemic reperfusion. **(E)** Samples were extracted for the R-7050 drug group, the ischemic group, the solvent group and the sham group to detect nSMase2 activity and protein expression levels. Data are presented as means ± SEM of four separate experiments (*n* = 4). *P < 0.05 vs. sham control. #*P* < 0.05 vs. ischemia solvent control.

Given that the internal cell TNF-α receptor-FAN-RACK1-EED-nSMase2 complex mediated by TNF-α stimulation could increase nSMase2 activity [27-29], coimmunoprecipitation technology was applied to detect the underlying mechanism associated with the different durations of I/R (0.5, 1, 6 and 24 h), and control experiments without the primary antibodies were performed to prove the specificity of the binding in the preliminary study. As the data in Figures 4A and B show, the combination of nSMase2/RACK1 and that of nSMase2/EED were augmented at I/R 30 min (*P* < 0.05), peaked at I/R 1 h (*P* < 0.05) and then gradually declined after I/R 24 h. Following treatment with the TNF-α receptor (TNF-αR) inhibitor R-7050 (10 μM), the combination of nSMase2/RACK1 or nSMase2/EED declined significantly in comparison to the solvent group (*P* < 0.05) (Figure 4C). Incidentally, nSMase2 activity was found to be partially reduced (*P* < 0.05) but remained significantly higher than that of the control group. There was no obvious variation in aSMase activity (*P* > 0.05) (Figure 4D). These results indicate that, in addition to the TNF-αR/RACK1/EED pathway, there might be other signals involved in ischemia-induced early initiation of nSMase2 in rat hippocampi.

### nSMase2 phosphorylation induced by p38MAPK is an important mechanism underlying nSMase2/ceramide pathway signaling during cerebral ischemia

**Figure 5:**
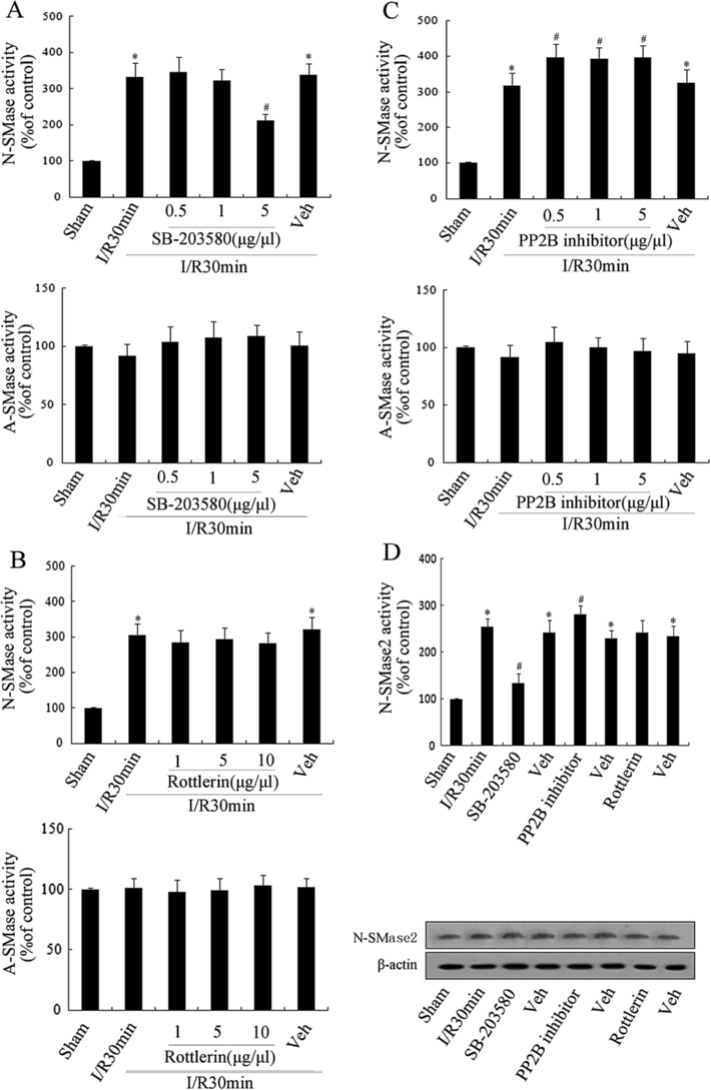
**Effect of p38 mitogen-activated protein kinase, protein phosphatase 2B or protein kinase Cδ inhibitors on initiation of neutral sphingomyelinase 2 following ischemia.** Some inhibitors and their solvents were separately injected into the lateral ventricle, followed by 30-min postischemic reperfusion. Effects of p38 mitogen-activated protein kinase (p38MAPK) inhibitor SB-203580 **(A)**, protein kinase Cδ (PKCδ) inhibitor rottlerin **(B)** and protein phosphatase 2B (PP2B) inhibitor on the activity levels of neutral sphingomyelinase 2 (nSMase2) and acid sphingomyelinase (aSMase) were examined using a sphingomyelinase assay kit **(C)**. **(D)** Protein expression and activity levels of nSMase2 in the rat hippocampus were detected in the different groups. Data are presented as the means ± SEM of four separate experiments (*n* = 4). *P < 0.05 vs. sham control. #*P* < 0.05 vs. ischemia solvent control.

Phosphorylation has been regarded as an important mechanism for nSMase2 activity. For example, p38MAPK, PKCζ and PP2B may regulate nSMase2 activity through phosphorylation [32-34]. To explore whether this underlying mechanism plays a key role in nSMase2 activity after cerebral ischemia, the p38MAPK inhibitor SB-203580, the PKCζ inhibitor rottlerin and the PP2B inhibitor were injected into the lateral ventricle, respectively. According to the data shown in Figures 5A, B and C, only SB-203580 could significantly inhibit nSMase activity in a dose-dependent manner (*P* < 0.05). To further investigate the effect of p38MAPK, PKCδ and PP2B on nSMase2 activity, the specificity of detection was examined after each inhibitor treatment (Figure 5D). SB-203580 was found to inhibit nSMase2 activity (*P* < 0.05), PP2B inhibitor enhanced its activity (*P* < 0.05) and rottlerin had little influence (*P* > 0.05). In addition, the nSMase2 protein content of each group appeared to be similar, implying that the difference was due to its own activity. nSMase2 phosphorylation induced by p38MAPK therefore appeared to play an important role in the rise of activity that occurred after cerebral I/R, whereas PP2B was linked to nSMase2 dephosphorylation and inactivation.

### A2B adenosine receptor regulates the initiation of nSMase2/ceramide pathway signaling stimulated by p38MAPK during cerebral ischemia

**Figure 6:**
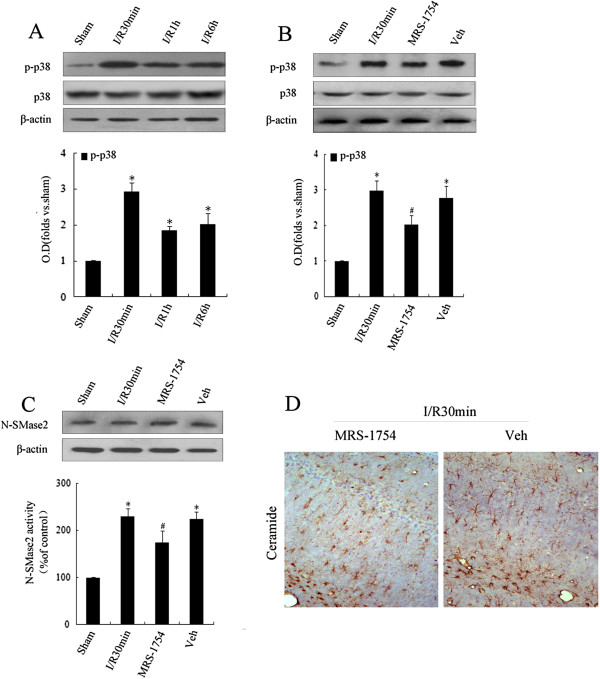
**Effect of the A2B adenosine receptor/p38 mitogen-activated protein kinase pathway on the early initiation of ischemia-induced neutral sphingomyelinase 2/ceramide. (A)** Phosphorylation levels of p38 mitogen-activated protein kinase (p38MAPK) were detected in the rat hippocampus after post-ischemia-reperfusion (post-I/R) of different durations (0.5 h, 1 h and 6 h). After A2B adenosine receptor inhibitor MRS-1754 or its solvent was injected into the lateral ventricle, 30-min post-I/R followed. **(B)** Protein expression and activity levels of neutral sphingomyelinase 2 (nSMase2) and **(C)** phosphorylation levels of p38MAPK in the rat hippocampus were detected in the different groups. **(D)** Content of ceramide in the rat hippocampus was determined using immunohistochemistry in MRS-1754 and its solvent groups. Data representat the means ± SEM of at least four separate experiments (*n* = 4). *P < 0.05 vs. sham control. #*P* < 0.05 vs. ischemia solvent control.

p38MAPK is an important member of the MAPK family which is involved in the regulation of cell differentiation, apoptosis and inflammation [46,47]. p38MAPK phosphorylation induced by A_2B_AR in gliomas can participate in the regulation of inflammation [35,36]. To clarify the possible involvement of A_2B_AR in p38MAPK phosphorylation, nSMase2 activation and ceramide production, the A_2B_AR inhibitor MRS-1754 (10 μg/μl) was administered following I/R. First, Western blot analysis showed that p38MAPK phosphorylation levels significantly increased after 30 min of I/R (*P* < 0.05) (Figure 6A) and subsequently decreased after 1 h and 6 h, but levels remained higher than those in the control group (*P* < 0.05). Second, MRS-1754 reversed the elevation of p38MAPK phosphorylation at 30 min (*P* < 0.05) (Figure 6B). In addition, MRS-1754 significantly inhibited nSMase2 activity (*P* < 0.05) (Figure 6C) but had no influence on aSMase activity (*P* > 0.05, data not shown). The immunohistochemical results revealed that ceramide levels were reduced in the rat hippocampi with the inhibition of A_2B_AR by MRS-1754 (Figure 6D). Taken together, the results suggest that A_2B_AR participated in the increment of nSMase2 activity induced by p38MAPK phosphorylation and the accumulation of ceramide during cerebral I/R.

### Neutral sphingomyelinase 2 involved in inflammation factor production in astrocytes following cerebral ischemia

**Figure 7:**
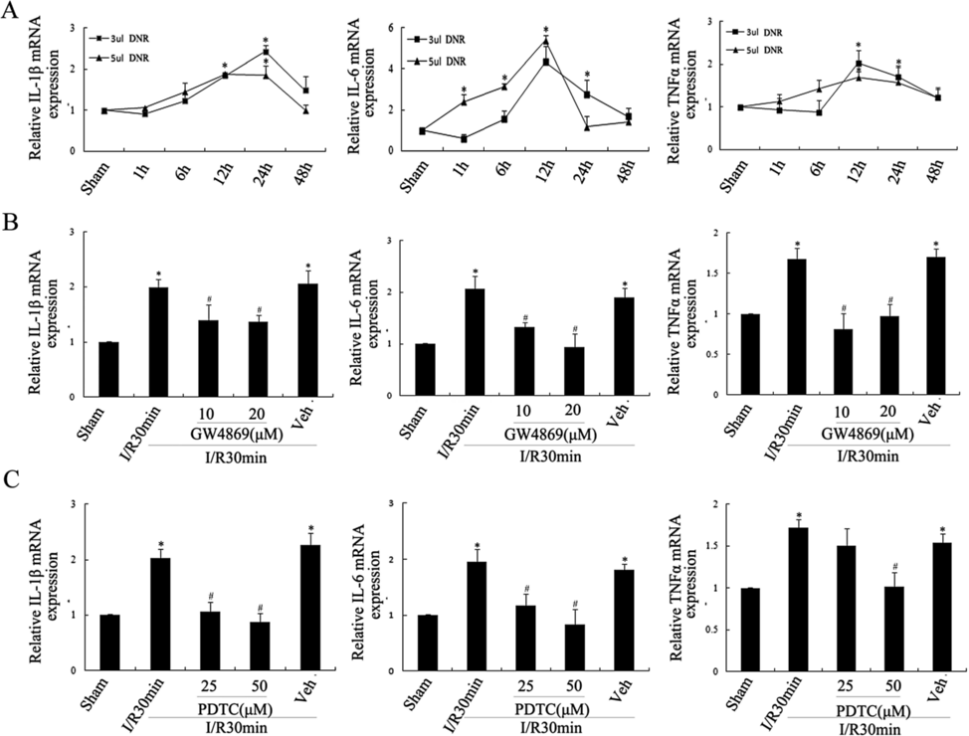
**Effect of the neutral sphingomyelinase 2/ceramide pathway on inflammation after cerebral ischemia.** Real-time polymerase chain reaction was performed to detect the mRNA levels of interleukin 1β (IL-1β), IL-6 and tumor necrosis factor α (TNF-α) after different treatments. **(A)** Sham group with daunorubicin (DNR) stimuli of different durations (1 h, 6 h, 12 h, 24 h and 48 h). Injection with GW4869 **(B)** or pyrrolidine dithiocarbamate (PDTC) **(C)** into the lateral ventricle prior to 30-min reperfusion postischemia. Data are presented as the means ± SEM of at least four separate experiments (*n* = 4). *P < 0.05 vs. sham control. #*P* < 0.05 vs. ischemia solvent control.

Oxidative stress and inflammation are important pathological factors in cerebral ischemic lesions [3,4]. Real-time PCR was used to detect the mRNA levels of inflammatory cytokines such as IL-1β, IL-6 and TNF-α associated with nSMase2 activation. After the nSMase2 agonist DNR was injected into the lateral ventricle, IL-6 mRNA levels started to rise at 1 h, peaked at 12 h and began to decline at 24 h (*P* < 0.05) (Figure 7A). The mRNA levels of IL-6 and TNF-α significantly increased at 12 h and did not decline until 24 h after treatment (*P* < 0.05) (Figure 7A). These data indicate that the activation of nSMase2 could drive the generation and release of inflammatory cytokines. To explore this hypothesis further, the nSMase2 inhibitor GW4869 and the nuclear factor κB (NF-κB) inhibitor pyrrolidine dithiocarbamate (PDTC) were injected into the rat hippocampus prior to ischemia, respectively. The real-time PCR findings (shown in Figures 7B and 7C) suggest that the inhibition of both nSMase2 and NF-κB activity could significantly reduce the mRNA levels of IL-1β, IL-6 and TNF-α (*P* < 0.05). Taken together, the activation of nSMase2 in astrocytes is suggested to have induced the production and release of IL-1β, IL-6 and TNF-α through NF-κB activity, thereby mediating the hippocampal neuronal damage that occurred during cerebral I/R.

### Ceramide accumulation in astrocytes is involved in damage of peripheral neurons following cerebral ischemia

**Figure 8:**
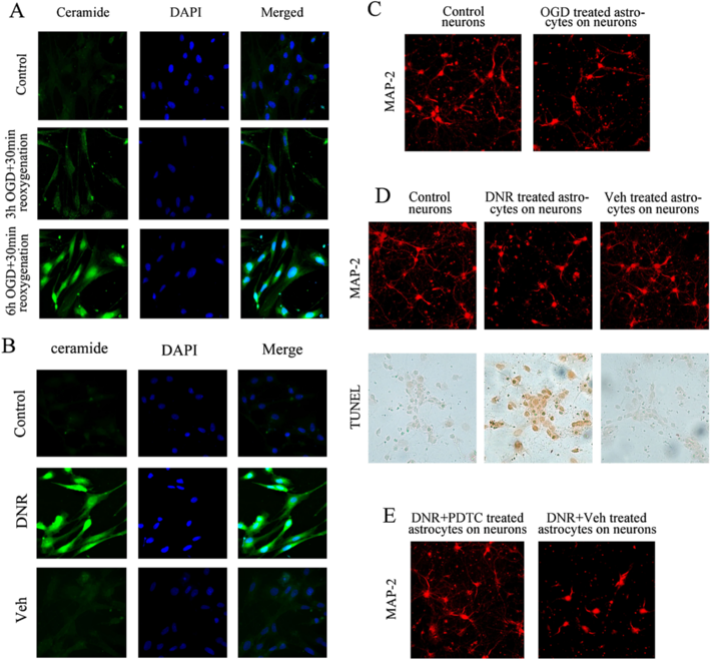
**Effect of in vitro oxygen-glucose deprivation in primary rat astrocytes on peripheral neuron injury.** The double-staining of ceramide and 4′,6-diamidino-2-phenylindole were detected by immunofluorescence in the different groups after primary rat astrocytes were deprived of oxygen-glucose in vitro for 3 h and 6 h, before regaining 30 min oxygen-glucose **(A)**, or were treated with daunorubicin (DNR) for 24 h **(B)**. **(C)** After primary rat astrocytes were deprived of oxygen-glucose in vitro for 6 h (before regaining 30-min oxygen-glucose), they were cocultured with primary rat neurons for 12 h and the latter were labeled with microtubule-associated protein 2 (MAP2) fluorescence. **(D)** After pretreatment with DNR for 24 h, astrocytes were cocultured with neurons for 12 h and then MAP2 and terminal deoxynucleotidyl transferase–mediated deoxyuridine triphosphate nick-end labeling staining were used to detect neuronal injury. **(E)** After concurrent pyrrolidine dithiocarbamate (PDTC) and DNR pretreatment, astrocytes were cocultured with neurons for 12 h and the latter were labeled with MAP2 fluorescence (*n* = 4).

To confirm the speculation that cerebral ischemia can induce peripheral neuronal damage through the nSMase2/ceramide pathway in astrocytes, an in vitro OGD model of rat primary astrocytes and a coculture model of primary astrocytes/primary neurons were established. All results in this study reflect activity at the cellular level. The findings clearly showed that ceramide content began to increase when primary astrocytes were deprived of oxygen-glucose in vitro for 3 h and then provided with both oxygen and glucose for 30 min. The increment was considerable when the length of deprivation reached 6 h (Figure 8A). In addition, astrocytes were treated with DNR (0.5 μM) for 24 h, which resulted in the generation of large amounts of ceramide compared with the control group and the solvent group (Figure 8B), indicating that DNR could induce ceramide generation by activating nSMase2. A cell coculture model, namely, neurons and astrocyte cells (OGD for 6 h and both oxygen and glucose for 30 min), was subsequently adopted to explore the relationship between ceramide accumulation and neuronal damage. This revealed that neuronal injury appeared with decreased MAP2 tags after coculture (0.78-fold vs. control; *P* < 0.05) (Figure 8C); DNR treatment in astrocytes induced the apoptosis of neurons, which was established using MAP2 labeling and a TUNEL assay (0.71-fold or 5.37-fold vs. vehicle, respectively; *P* < 0.05) (Figure 8D); and PDTC significantly reversed this situation when used in combination with DNR, established using a MAP2 labeling assay (1.87-fold vs. vehicle; *P* < 0.05) (Figure 8E). Collectively, these results suggest that cerebral ischemia can induce the activation of nSMase2 in astrocytes to generate ceramide and then mediate the secondary damage of neurons through an inflammatory response regulated by NF-κB.

## Discussion

Over the past several decades, increasing attention has been paid to the effects of neuronal activities in stroke research [48,49]; however, these efforts have failed to provide a stroke treatment [50,51]. To improve the chances of finding an effective stroke treatment, a broader focus on the loss and dysfunction of non-neuronal cell types is required. Of the several glial cell types, astrocytes are the most abundant cell type and play key roles in the physiology and pathology of the central nervous system [52,53]. In the present study, ceramide, which is a risk factor for neuronal damage, was found to be accumulated in the astrocytes, but not in the neurons, of the rat hippocampus after ischemia.

Ceramide, which is a lipid second messenger, is known to be involved in neuronal damage through intracellular signaling pathways in response to harmful stimuli [9,10,44]. However, the role of ischemia-induced ceramide production in astrocytes is poorly understood. In the present study, the ceramide signal was found to be related to a variety of proinflammatory cytokines, including TNF-α, IL-1β and IL-6 production, as a result of NF-κB signaling. First, ceramide accumulation induced by an nSMase2 agonist [45] could effectively stimulate the increment of proinflammatory cytokine mRNA levels in the hippocampi. Second, the reduction of ceramide levels by an nSMase2 inhibitor could attenuate mRNA levels of proinflammatory cytokines in the hippocampus following ischemia, suggesting that ceramide signals are closely associated with neuroinflammation in the rat hippocampi in response to forebrain ischemia, which is similar to findings in previous reports [44,54-57]. Third, the inactivation of NF-κB signaling could decrease the production of proinflammatory cytokines (TNF-α, IL-1β and IL-6) in response to ceramide signaling, suggesting that the ceramide-induced upregulation of proinflammatory cytokine is dependent on NF-κB signaling.

Inflammation induced by ceramide in astrocytes was found to be closely associated with neuronal damage following cerebral ischemia. The data from the Transwell experiments suggest that ceramide accumulation in the astrocytes can result in primary neuron damage in response to OGD and activation of the nSMase2/ceramide pathway, which could be reversed by inhibiting NF-κB signaling. Inflammation-related cytokine release from activated astrocytes is thought to be characteristic of some neurodegenerative diseases, such as Alzheimer disease, which is associated with progressive neuronal damage [37,38,58-60]. Therefore, the present study hypothesized that activated astrocytes might be involved in the damage and loss of neurons in rat hippocampi as a result of ceramide-induced neuroinflammation following transient forebrain ischemia.

How is ceramide signaling controlled after cerebral ischemia? The present study has highlighted the importance of nSMase2 in the ceramide accumulation that occurred in the rat hippocampus following cerebral ischemia, which was activated especially within astrocytes [44,52,53]. nSMase activity was upregulated after cerebral ischemia, in accordance with an early accumulation of ceramide. Meanwhile, the downregulation of nSMase2 by either a chemical inhibitor or siRNA reduced ceramide production in hippocampal and primary astrocytes. However, the inhibition of aSMase had no effect on the ischemia-induced activation of astrocytes, emphasizing the possible specificity of the effect. Previous studies have demonstrated the involvement of nSMase2 in astrocyte ceramide accumulation in response to the stimulation of fibrillar amyloid-β peptide [44]. The present study also suggests that the inhibition of nSMase2 could effectively attenuate the expression of proinflammatory cytokines in ischemia-stimulated astrocytes (associated with ceramide signaling). Therefore, the inhibition of nSMase2 in the astrocytes could also partly reverse the neuronal damage that occurred in response to cerebral ischemia. Furthermore, the cellular localization of nSMase2 in astrocytes but not in neurons supports its association with ceramide production. The data indicate that nSMase2 plays a key role in ischemia-induced ceramide accumulation and in its function within rat hippocampal astrocytes.

nSMase2 can be activated by TNF-α stimuli through the binding of nSMase2 to TNF-αR/RACK1/EED and is important for inflammatory signaling [27-29]. In the present study, coimmunoprecipitation data suggest that cerebral ischemia induced the increased binding of nSMase2 with RACK1 and EED, which might have been associated with nSMase2 activation in the early phase of ischemia. However, the inhibition of TNF-αR attenuated the nSMase2 activity to some extent, suggesting that the TNF-αR/RACK1/EED pathway plays a secondary role in the upregulation of nSMase2 activity in hippocampal astrocytes following ischemia. Meanwhile, TNF-α has been reported to upregulate aSMase activity and subsequently modulate NF-κB-dependent inflammatory signaling [61,62], but the ischemia-induced activation of SMase is not linked to aSMase. The data in the current study suggest that ischemia-induced nSMase2 activation might have been partly dependent on the TNF-αR signaling pathway. Further investigation is required to examine other possible mechanisms underlying nSMase2 activation.

Phosphorylation plays a crucial role in nSMase2 activity [32-34]. In the present study, p38, but not PKCζ or PP2B, was found to be involved in nSMase2 activation in the rat hippocampi following ischemia. First, cerebral ischemia induced the rapid upregulation of p38 activity, in accordance with nSMase2 activation at 30 min post-I/R. Second, the p38 inhibitor could reverse the upregulation of nSMase2 and reduce ceramide levels in response to ischemia. Previous studies have demonstrated that p38 can result in nSMase2 activation through the phosphorylation of its special site and that it is associated with inflammation stress [35]. Furthermore, the A_2B_AR inhibitor can also result in downregulation of nSMase2 activity and ceramide levels, which are closely linked to p38 dephosphorylation. It has been reported that A_2B_AR plays a key role in the rapid activation of p38 and the subsequent upregulation of inflammation. Although there is controversy regarding whether the effects of A_2B_AR are harmful or beneficial, A_2B_AR is widely thought to be involved in the inflammatory response [35,36]. p38, nSMase2 and ceramide signaling are closely associated with the upregulation of inflammatory factors. Therefore, this study supports the viewpoint that A_2B_AR/p38 has a crucial role in the activation of the nSMase2/ceramide pathway and the underlying inflammation in rat hippocampi in response to ischemia.

## Conclusions

**Figure 9:**
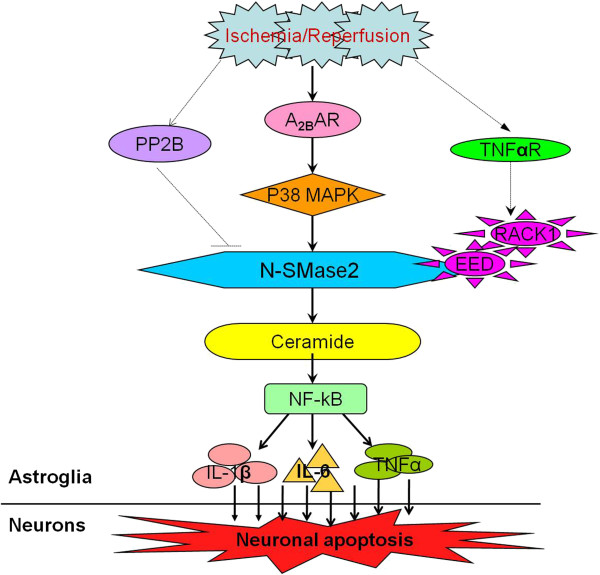
**Process flow diagram of signaling pathway.** Specific accumulation of ceramide in astrocytes can be induced by cerebral ischemia-reperfusion (I/R), of which activation of neutral sphingomyelinase 2 (nSMase2) by the A2B adenosine receptor (A_2B_AR)/p38 mitogen-activated protein kinase (p38MAPK) pathway is the major mechanism for ceramide production. Meanwhile, the binding of the TNF-αR-RACK1-EED complex via their WD repeat domains with nSMase2 can initiate its activity only in part. More importantly, the ischemia-induced accumulation of ceramide in astrocytes can upregulate the generation of proinflammatory factors such as interleukin 1β (IL-1β), IL-6 and tumor necrosis factor α (TNF-α) mediated by nuclear factor κB (NF-κB), leading to secondary damage of hippocampal neurons.

The results of this study reveal that cerebral ischemia induced the activation of the nSMase2/ceramide pathway in astrocytes, but not neurons in the rat hippocampus. This involved the upregulation of preinflammation signaling and neuronal damage resulting from a neuroinflammation mediator. However, nSMase2 activation was associated with the TNF-αR/RACK1 pathway, and ischemia-induced A_2B_AR upregulation and p38 activation played a key role in nSMase2/ceramide pathway signaling (Figure 9). These data highlight the need to unravel the mechanisms of ceramide signaling in activated astrocytes and astrocyte-mediated neuronal damage resulting from neuroinflammation. Such information would provide significant insight into the pathophysiology of cerebral ischemia and aid the development of treatment paradigms.

## Abbreviations

As: Astrocyte; A2BAR: A2B adenosine receptor; aSMase: Acid sphingomyelinase; CA: Cornu ammonis; Cera: Ceramide; DG: Dentate gyrus; DNR: Daunorubicin; FAN: factor associated with neutral sphingomyelinase activation; FBS: Fetal bovine serum; HRP: Horseradish peroxidase; IL: Interleukin; IP: Immunoprecipitation; I/R: Ischemia/reperfusion; MAPK: Mitogen-activated protein kinase; NeuN: Neuronal nuclei; nSMase: Neutral sphingomyelinase; OGD: Oxygen-glucose deprivation; PKC: Protein kinase C; PP: Protein phosphatase; RACK1: receptor for activated C kinase 1; RT: Reverse transcription; siRNA: Small interfering RNA; TNF: Tumor necrosis factor; TUNEL: Terminal deoxynucleotidyl transferase–mediated deoxyuridine triphosphate nick-end labeling; VO: Vessel occlusion.

## Competing interests

The authors declare that they have no competing interests.

## Authors’ contributions

LZG conducted all experiments. BSH and WS conducted experiments and participated in discussion and analysis of data. LG prepared the first draft of the manuscript. ZZD carried out the immunohistochemical staining. HWW and JG designed the project and finalized the manuscript. All authors read and approved the final manuscript.
